# *Ex vivo* analysis of topotecan: advancing the application of laboratory-based clinical therapeutics

**DOI:** 10.1038/sj.bjc.6601336

**Published:** 2003-10-28

**Authors:** R A Nagourney, B L Sommers, S M Harper, S Radecki, S S Evans

**Affiliations:** 1 Rational Therapeutics, Inc., 750 East 29th Street, Long Beach, CA 90806, USA; 2 Malcolm C. Todd Cancer Institute, 2801 Atlantic Avenue, Long Beach, CA 90806, USA; 3 College of Medicine at University of California, Irvine

**Keywords:** topotecan, combination regimens, non-small-cell lung cancer, breast cancer, cell-death assay, *ex vivo*

## Abstract

Topotecan is currently approved for relapsed small-cell lung cancer and ovarian cancer. Topotecan's efficacy in the second-line setting and novel mechanism of action suggest broad antitumour activity. We utilised a clinically validated, cell-death, *ex vivo* assay in human tumour explants to examine the activity profile of topotecan alone and in combination with other antitumour agents. Serial dilutions of topotecan alone and in combination with other cytotoxic agents were applied to biopsy specimens of non-small-cell lung cancer (NSCLC) and breast, colon, and prostate cancers. Dose–response curves were interpolated to provide 50% lethal concentrations (LC_50_). The degree of synergy (by median effect) and normalised *Z*-scores (raw scores converted to relative activity distributed around the mean) were then computed. Single-agent activity was observed for topotecan in all four tumour types. In 57 chemotherapy-naive specimens, NSCLC revealed the highest activity, demonstrated by the lowest LC_50_ value (0.26±0.06 *μ*g ml^−1^; *P*=0.002). Overall, previously treated and chemotherapy-naive specimens revealed no significant differences in mean LC_50_'s. Synergy was observed for several combinations, including topotecan plus cisplatin in prostate and for topotecan plus 5-fluorouracil in breast cancers. The *Z*-score analyses conducted suggest activity for previously unexplored drug regimens, including topotecan plus 5-fluorouracil, vinorelbine, and mitomycin-C in NSCLC and breast cancer. Phase II studies are underway to determine the degree to which these *ex vivo* findings will translate into improved clinical results.

Topotecan, a semisynthetic, water-soluble derivative of camptothecin, is an inhibitor of topoisomerase I (Kingsbury *et al*, 1991). This derivative is more stable, has increased solubility, a shorter half-life, and decreased toxicity compared with its parent compound (Verweij *et al*, 1993; Burke and Mi, 1994; Rothenberg, 1997). Topoisomerase I is a nuclear enzyme that relieves torsional strain on supercoiled DNA and creates single-strand breaks during DNA replication. Topotecan prevents topoisomerase I from repairing the cleaved DNA, which results in double-stranded DNA breaks and eventually apoptosis. The unique mechanism of action of topotecan and lack of clinical crossresistance with other antineoplastic compounds suggest that topotecan has the potential for broad antitumour activity.

The activity of topotecan has been demonstrated in several open-label, randomised phase II and III trials. Currently, topotecan is approved for the treatment of relapsed small-cell lung cancer (SCLC) (Eckardt *et al*, 1996; Ardizzoni *et al*, 1997; Depierre *et al*, 1997; von Pawel *et al*, 1999,^2001^) and ovarian cancer (Creemers *et al*, 1996; Kudelka *et al*, 1996; ten Bokkel Huinink *et al*, 1997; Bookman *et al*, 1998; Hoskins *et al*, 1998; McGuire *et al*, 2000). Although topotecan is typically given in a 30-min intravenous (i.v.) infusion on days 1–5 of a 21-day course, other clinical schedules have been investigated, including 21-day continuous i.v. infusion (Hochster *et al*, 1999), 3-day administration (Brown *et al*, 2000), single-day administration every 21 days (Wall *et al*, 1992), weekly bolus (Homesley *et al*, 2001), and weekly 24-h continuous i.v. infusion (Hoskins *et al*, 1998). In addition, an oral formulation of topotecan has also been investigated in SCLC (von Pawel *et al*, 2001) and ovarian cancer (Clarke-Pearson *et al*, 2001). Preliminary reports indicate that topotecan also has activity in non-small-cell lung cancer (NSCLC) (Lynch *et al*, 1994; Perez-Soler *et al*, 1996), cervical cancer (Fiorica *et al*, 2002), and haematologic malignancies (Beran *et al*, 1998). Additional studies are required to determine the activity of topotecan in other cancers.

Topotecan activity also suggests potential for synergy in combination with other cytotoxic agents. The predictable toxicity profile of topotecan makes it feasible to combine topotecan with other agents with nonoverlapping toxicities. Topotecan has been investigated in doublet and triplet combinations with several agents, including cisplatin, paclitaxel, and etoposide (Ardizzoni *et al*, 1999; Frasci *et al*, 1999; Hainsworth *et al*, 1999; Jacobs *et al*, 1999; O'Neill *et al*, 2001; Fiorica *et al*, 2002). Randomised trials are underway in several diseases to evaluate the efficacy of topotecan in combination with other agents.

*Ex vivo* analyses of human tumour primary cultures can provide disease-specific drug activity profiles and powerful insights useful in the development of phase I/II trials. The present study applied an *ex vivo* assay, based on the delayed loss of membrane integrity, to investigate the clinical potential of topotecan in human tumour biopsy specimens other than relapsed SCLC and ovarian cancer. Although earlier laboratory assays based on drug-induced growth inhibition (e.g. H_3_*-thymidine incorporation, clonogenic) have consistently failed to provide clinically relevant information, newer methods that incorporate cell-death measures as surrogates of drug-induced apoptosis have been shown to correlate with response, time to progression, and survival in human haematologic and solid tumours (Bosanquet *et al*, 1999; Cortazar and Johnson, 1999; Nagourney *et al*, 2000,^2003^). The realisation that most chemotherapeutic agents exert their cytotoxic effects through apoptosis (Reed, 1999) supports the use of cell-death assay in the study of topotecan's antitumour activity in human neoplasms.

The principal focus of this study was the investigation of topotecan activity in biopsies of previously untreated NSCLC and breast, colorectal, and prostate carcinomas. We also assessed the synergy of topotecan in combination with other antineoplastic agents and compared the activity of topotecan in chemotherapy-naive *vs* previously treated specimens. Additional analyses compared the activity of topotecan with that of irinotecan, the related topoisomerase I inhibitor, in parallel studies.

## MATERIALS AND METHODS

### Tissue procurement and tumour-cell preparation

Patients undergoing surgical procedures for the management of NSCLC and breast, colorectal, or prostate cancers were offered the opportunity to participate in the study. All patients provided written informed consent allowing the release of tumour biopsy specimens for laboratory analysis. At the time of surgical exploration, tumour tissues removed from the patients were processed sterilely by the Department of Pathology at the Long Beach Memorial Medical Centre (Long Beach, CA, USA). After histologic diagnostic evaluations, the tissue samples were placed in sterile RPMI 1640 media containing 2 mM L-glutamine, 15% fetal calf serum, 100 IU ml^−1^ penicillin, and 100 *μ*g ml^−1^ streptomycin (modified RPMI 1640), and were submitted directly to our laboratory. Samples obtained after hours were maintained at 4°C until processed (always <24 h). This study was approved by the Long Beach Memorial Medical Centre Human Subjects Committee.

Tumour specimens were mechanically disaggregated by sterile mincing with scalpels. Specimens were then incubated for 2 h in 0.8% collagenase IV and 0.002% *DNase*I. Cells were isolated by density centrifugation over Ficoll–Hypaque, washed, and then resuspended in modified RPMI 1640. Cell suspensions were adjusted to 1 × 10^6^ cells ml^−1^ and distributed into 96-well polypropylene culture plates (90 *μ*l well^−1^). Serial dilutions of drugs and drug combinations in 10 *μ*l volumes were added, and the plates were incubated at 37°C in 6% CO_2_ in a sterile, humidified environment for 72 h as described in the next section.

### Drug exposure and measurement of cell viability

The dose-dependent cytotoxicity of drugs was investigated using a five-point dose–response curve. Serial dilutions of cytotoxic drugs were prepared in 0.15 M NaCl, and 10 *μ*l of a drug solution was added to each well. Cells were incubated for 72 h with saline vehicle control (0.15 M NaCl) or in the presence of topotecan (range, 0.03–0.92 *μ*g ml^−1^), alone or in combination at a fixed ratio in serial dilution with 5-fluorouracil (5-FU; range, 3.1–100 *μ*g ml^−1^), cisplatin (range, 0.2–6.6 *μ*g ml^−1^), doxorubicin (range, 0.04–1.2 *μ*g ml^−1^), gemcitabine (range, 8.2–263 *μ*g ml^−1^), mitomycin-C (range, 0.04–1.2 *μ*g ml^−1^), mitoxantrone (range, 0.03–1.0 *μ*g ml^−1^), nitrogen mustard (range, 0.17–5.4 *μ*g ml^−1^), oxaliplatin (range, 0.3–10 *μ*g ml^−1^), paclitaxel (range, 1.6–50 *μ*g ml^−1^), or vinorelbine (range, 0.3–10 *μ*g ml^−1^). After 72 h, 10 *μ*l of phosphate-buffered saline containing 0.5% nigrosin-B and 1% Fast Green and 37 500 glutaraldehyde-fixed avian red blood cells (internal standard) were added to each well, and the samples were gently agitated to facilitate mixing. After 10 min, samples were aspirated and centrifuged onto a glass slide using a modified cytospin cassette. Air-dried samples were counterstained with modified haematoxylin and eosin. Tumour cell viability was determined as the ratio of living tumour cells over simultaneously counted avian red blood cells. Cell survival of drug-treated samples was expressed as a percentage of saline control values.

### Calculation of LC_50_, synergy, and *Z*-score

The 50% lethal concentration (LC_50_) values were calculated using a least-squares line of best-fit generated from the five-point concentration curve, with the LC_50_ values interpolated from the curve. Synergy was determined using the median effect technique (Chou and Talalay, 1987, pp 37–64). In brief, dose–response curves and LC_50_ values were generated for topotecan both as a single agent and in combination with other agents. Comparison of the topotecan single-agent dose–response curves with the combination dose–response curves for a given tumour type allowed the determination of synergy, additivity, subadditivity, or antagonism. The term *synergy* only applied to tumour samples with 100% of the points on the combination dose–response curves falling above the line of additivity, while *partial synergy* applied to samples for which >50% of the points on the dose–response curve were above the line of additivity.

The overall mean LC_50_ (LC_50_T) and s.e.m. for all tumour types (s.e.m T) within our laboratory database were utilised to calculate *Z*-scores by the following formula:


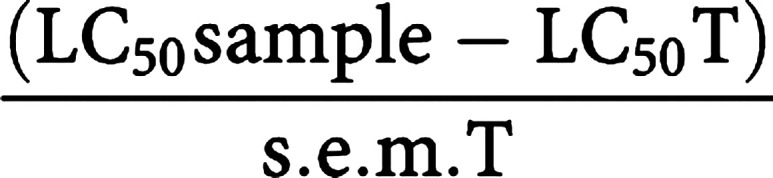


The use of inferential statistics is predicated upon the central limit theorem (Feinstein, 1998), a fundamental sampling theorem that states that the distribution of a sample population is unimportant so long as the samples are drawn randomly in sufficient numbers from a parent population whose distribution need not have a normal (Gaussian) distribution (Hays, 1994).

By normalising the LC_50_ values for each single agent and/or combination around the mean LC_50_ values, samples found to be more resistant than average fell to the right of the mean, whereas those that were more sensitive than average fell to the left of the mean. This *Z*-score transformation is routinely used in the US by the National Cancer Institute for studies comparing microarray data and other analyses (Cheadle *et al*, 2003). It has been incorporated into the COMPARE statistical program (Shi *et al*, 1998), and into the latest version of the National Cancer Institute's public access MAExplorer bioinformatics tool (Lemkin *et al*, 2000).

## STATISTICAL METHODS

Statistical analysis of the study data was carried out using the analysis of variance program from the Statistical Package for the Social Sciences (Levine, 1991). The LC_50_ means for single-agent topotecan in untreated biopsies were compared across tumour types, with the criterion for statistical significance set at *P*⩽0.05. For comparisons of *in vitro* activity of topotecan in combination with other agents, LC_50_ values were normalised into *Z*-scores (with the mean set to 0 and variance from the mean in s.e.m. units of 1) (Kirk, 1982). This permitted direct comparisons of the sensitivity *vs* resistance of drug combinations across tumour types. Using these normalised LC_50_ values, graphic representations were generated depicting on the left side of the mean those samples more sensitive than average to drug combinations (negative range), and on the right side of the mean those more resistant than average to drug combinations (positive range). Finally, the activity of topotecan *vs* irinotecan in tumour specimens was assessed using a scatter gram graph and was statistically compared using the Pearson product-moment correlation from the same statistical package.

## RESULTS

### Activity of single-agent topotecan

In aggregate, our topotecan database includes a total of 697 analyses, of which 380 are from NSCLC, and breast, colon, and prostate cancer biopsies that have been studies against single-agent topotecan. These include 149 specimens from chemotherapy-naive patients (untreated specimens) and 231 specimens from previously treated patients (previously treated specimens).

**Figure 1:**
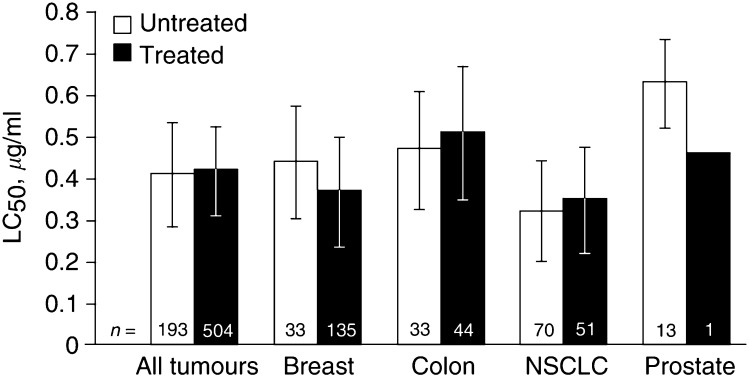
LC_50_ values for single-agent topotecan in tumour biopsies taken from untreated and previously treated patients in the master database. Tumour biopsies from patients with breast cancer, colon cancer, NSCLC, or prostate cancer were incubated with various concentrations of topotecan, and the LC_50_ values were calculated. The numbers in parentheses indicate the number of samples for the indicated drug combination.

To assess the effect of prior chemotherapy exposure on the activity of topotecan, the LC_50_ values of topotecan in cells obtained from previously treated specimens were compared with those from untreated specimens (Figure 1

). The mean LC_50_ value for all previously treated specimens was only marginally higher than that for the untreated specimens. However, when comparing previously treated LC_50_ values with untreated LC_50_ values within tumour types, several trends emerged. For instance, a comparison between the LC_50_ values of 135 previously treated breast cancer specimens with 33 untreated breast cancer specimens indicated a trend toward greater sensitivity in the previously treated specimens. The opposite trend was observed in colon cancer and NSCLC specimens. By rank order, previously untreated NSCLC specimens exhibited the lowest LC_50_ value (*P*=0.002), whereas previously untreated prostate cancer specimens had the highest LC_50_ value, suggesting that NSCLC and breast cancer might be better targets for topotecan than colon or prostate cancers.

To address concerns that prior exposure to chemotherapy might pose a confounding variable, we procured 86 tumour specimens from previously untreated patients to conduct formal drug response and combination analyses. Viable tumour cells were successfully isolated from 74 (86%) of these specimens. Of 74 specimens, 57(77%), including 14 NSCLC and 18 breast, 13 prostate, and 12 colon cancer specimens, provided adequate cell yield for complete single-agent and a combination-agent analyses. Of the 74 specimens, 17(23%) provided adequate cell yield for partial analyses. The LC_50_ value (±s.e.m.) for single-agent topotecan in all 57 tumours was 0.43±0.04 *μ*g ml^−1^. The LC_50_ values for each tumour type are shown in Table 1

**Table 1:** LC_50_ (*μ*g ml^−1^) values for single-agent topotecan in untreated biopsies by tumour type

	**All tumours, *n*=57**	**Breast, *n*=18**	**Colon, *n*=12**	**NSCLC, *n*=14**	**Prostate, *n*=13**
Mean±s.d.	0.43±0.30	0.40±0.25	0.47±0.31	0.26±0.22*	0.63±0.22
					
Median	0.35	0.30	0.40	0.20	0.50

LC_50_=50% lethal concentration; NSCLC=non-small-cell lung cancer; s.d.=standard deviation.

* *P*<0.002. (*t*-test, comparing NSCLC to other tumour types).

. The NSCLC samples demonstrated the lowest LC_50_ value at 0.26±0.06 *μ*g ml^−1^ (*P*=0.002), suggesting that NSCLC is a potential target for topotecan treatment.

### Synergy of topotecan combinations

The mechanism of action of topotecan suggests that combinations with other cytotoxic agents may provide increased antitumour activity. To assess this potential, a variety of topotecan–drug combinations were investigated for activity and degree of synergy. The LC_50_ values for topotecan in combination with other agents, at fixed drug ratios, and the degree of synergy for the different drug combinations by tumour type are provided in Table 2

**Table 2:** *In vitro* activity of topotecan in combination with other agents

**Diagnosis (*n*)**	**Mean LC_50_ (*μ*g ml^−1^**)	Synergy*(%)	**Z-score**
*5-fluorouracil*			
Breast (9)	13.21	57	−1.70
Colon (12)	18.37	15	+0.46
NSCLC (3)	25.00	0	+3.25
			
*Cisplatin*			
Breast (9)	1.44	13	+2.31
Colon (11)	1.67	17	+4.17
NSCLC (20)	0.88	31	−2.28
Prostate (4)	0.88	75	−2.32
			
*Doxorubicin*			
Breast (17)	0.34	25	−1.76
Prostate (14)	0.61	25	+0.082
			
*Gemcitabine*			
Breast (10)	51.10	0	−1.32
Colon (11)	65.18	27	+1.25
NSCLC (18)	45.61	7	−2.32
Prostate (10)	81.0	0	+4.13
			
*Mitomycin-C*			
Colon (10)	0.96	40	+3.88
NSCLC (16)	0.45	38	−2.43
			
*Mitoxantrone*			
Breast (9)	0.32	17	−1.10
Prostate (8)	0.46	25	+1.24
			
*Nitrogen mustard*			
Breast (17)	1.11	25	+0.24
NSCLC (3)	0.97	0	−0.98
Prostate (4)	1.05	25	−0.28
			
*Oxaliplatin*			
Colon (10)	2.59	22	+0.76
NSCLC (16)	1.53	8	−3.13
Prostate (10)	3.52	20	+4.18
			
*Paclitaxel*			
Breast (13)	6.73	33	−0.38
NSCLC (19)	7.98	0	+1.59
			
*Vinorelbine*			
Breast (13)	2.04	25	−1.36
Colon (11)	2.96	27	+2.76
NSCLC (19)	1.82	13	−2.33
Prostate (10)	3.05	25	+3.15

* Defined as the percentage of tumour samples that demonstrated 100% synergy as described in Materials and Methods section.

LC_50_=50% lethal concentration; NSCLC=non-small-cell lung cancer.

. It is worth noting that the degree of synergy varied by drug combination and tumour type.

### *Z*-scores of topotecan combinations

**Figure 2:**
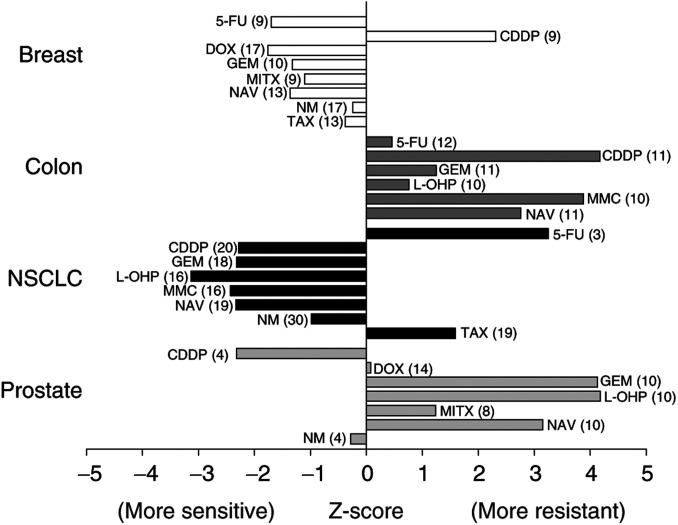
Z-scores of topotecan combination regimens in four tumour types. Z-scores of topotecan in biopsies from patients with breast cancer (open), colon cancer (dark grey), NSCLC (black), and prostate cancer (light grey). The numbers in parentheses indicate the number of samples for the indicated drug combination. NSCLC=non-small-cell lung cancer; 5-FU=5-fluorouracil; CDD*P*=cisplatin; DOX=doxorubicin; GEM=gemcitabine; MITX=mitoxantrone; NAV=vinorelbine; NM=nitrogen mustard; TAX=paclitaxel; L-OH*P*=oxaliplatin; MMC=mitomycin-C.

*Ex vivo* drug concentrations were based on the clinically correlated database. Concentration ranges, by drug, varied by as much as three orders of magnitude. To compare antitumour activity in highly disparate concentration ranges directly, raw LC_50_ values were normalised using *Z*-scores, as summarised in Table 2 and illustrated in Figures 2

and 3

**Figure 3:**
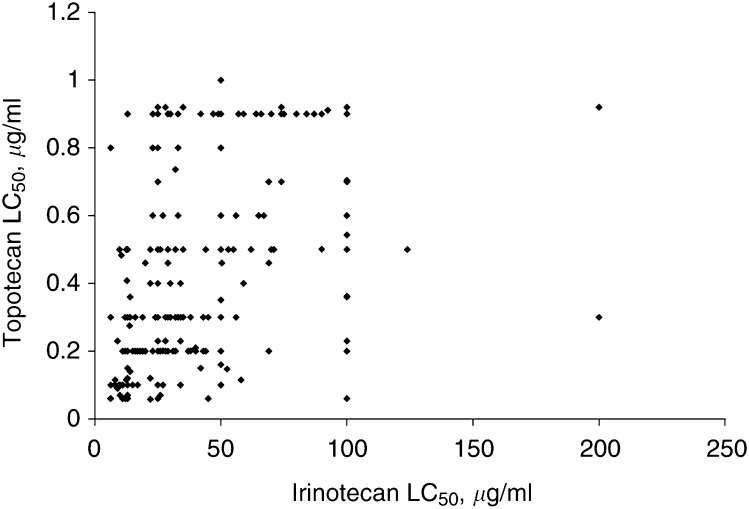
Relative *in vitro* activity of topotecan *vs* irinotecan. A scatterplot of the 50% lethal concentration values for topotecan and irinotecan in 239 tumour biopsy samples. A correlation coefficient of 0.54 indicates significant correlation between the antitumour activity of topotecan and irinotecan *in vitro* (*P*<0.0005).

. The data used in the generation of the s.e.m. values were obtained in previously untreated solid tumours in the laboratory database. These included NSCLC, colon, gastric, breast, ovarian, pancreatic, and uterine cancer specimens, ranging in number from 17 (mitoxantrone) to 53 (vinorelbine) with a median of 35 analyses per drug combination. A variety of combination regimens fell within the sensitive range of *Z*-scores for breast cancer and NSCLC tumour samples. The combination of doxorubicin and topotecan revealed significantly different *Z*-scores between breast and prostate cancer (*P*<0.001). Likewise, a comparison of prostate cancer, colon cancer, and NSCLC samples revealed significant differences in *Z*-scores across these tumour types for the combination of topotecan and oxaliplatin (*P*<0.01). Analyses of variance conducted across the four tumour types revealed significant differences in *Z*-scores for the topotecan/cisplatin combination (*P*<0.05). Finally, the topotecan/mitomycin-C combination resulted in significantly different *Z*-scores between NSCLC and colon cancer tumour samples (*P*<0.01). Only two combination regimens (topotecan/cisplatin and topotecan/nitrogen mustard) demonstrated *Z*-scores in the sensitive range in prostate cancer samples. None of the drug combinations tested on colon cancer samples had *Z*-scores within the sensitive range.

### Topotecan *vs* irinotecan

A unique advantage of these *ex vivo* analyses is their ability to compare drugs in parallel studies conducted on individual patient tumour specimens. Irinotecan, another topoisomerase I inhibitor, is a prodrug activated by carboxylesterase to form the active metabolite SN38. While spontaneous degradation or activation by carboxylesterases present in culture media is known to occur, and provides cytotoxic activity *in vitro*, some variability between irinotecan and topotecan would be expected based on topotecan's direct cytotoxic actions. When we directly compared the activity of topotecan against that of irinotecan in 239 chemonaive and previously treated human tumour specimens, there was a significant correlation between the LC_50_ values for topotecan *vs* irinotecan (*P*<0.0005). At the time of initial calculation, a portion of the LC_50_ values were rounded to the nearest 0.1 *μ*g ml^−1^ value.

## DISCUSSION

The clinical efficacy and favourable safety profile of topotecan in the treatment of relapsed SCLC and ovarian cancer patients, and topotecan's novel mechanism of action have prompted investigations into its use in other solid tumours. *Ex vivo* analyses, based on clinically validated end points, can identify disease-specific drug activity profiles and offer a rational approach to drug development. We applied *ex vivo* analyses to evaluate the activity of single-agent topotecan and topotecan combinations in human NSCLC and breast, colon, and prostate cancer specimens.

*Ex vivo* topotecan activity was highest for NSCLC and breast cancer specimens and lowest for prostate and colon cancer specimens. This suggests that NSCLC may be more sensitive to single-agent topotecan than prostate cancers.

Several phase II trials have investigated the use of topotecan in the treatment of advanced NSCLC. Perez-Soler *et al* (1996) and Weitz *et al* (2000) reported overall response rates of 15 and 16%, respectively, in patients treated with topotecan, and Lynch *et al* (1994) reported stable disease in 55%. In contrast, and consistent with the results of the current study, single-agent topotecan has shown less promise in patients with metastatic, hormone-refractory prostate cancer (Hudes *et al*, 1995). The synergy results for prostate and other cancers may nonetheless offer fertile avenues for future investigations with topotecan. A possible explanation for the difference between *in vitro* and *in vivo* activity of single-agent topotecan in NSCLC is a pharmacokinetic effect. The inherent activity (reflected *ex vivo*) for topoisomerase I inhibitors may not be optimally exploited using current administration schedules. Recent observations with alternative schedules – including oral, long-term i.v., or the weekly administration of topotecan – suggest that these regimens may prove superior to the current 5-day topotecan schedules in those diseases that are most inherently sensitive to topotecan, with NSCLC appearing to be an attractive target.

In our study, several topotecan combinations demonstrated synergy. Among the most active combinations was that of topotecan plus 5-FU in breast cancer, which demonstrated synergy in 57% of specimens. It must be noted that the degree of synergy (combinatorial advantage) is independent of the relative antitumour activity (i.e. LC_50_). However, synergy, defined as pharmacologic supraadditivity, may occur at LC_50_ values that have no clinical relevance. Therefore, it is advantageous to examine the degree of synergy in the context of the relative activity, and *Z*-scores facilitate this comparison. The *Z*-score for topotecan/5-FU in breast cancer fell in the sensitive range. Based on both synergy and sensitivity (by *Z*-score) findings for this combination in breast cancer patients, a phase II clinical trial has been initiated (phase II study of topotecan plus capecitabine for recurrent, locally advanced or metastatic breast cancer). This study will examine the clinical activity of this combination and correlate clinical response with *ex vivo* results for each patient.

To date, breast cancer has not been a target for topotecan therapy. Levine *et al* (1999) reported a response rate of 10% in a study of topotecan in relapsed breast cancer conducted by the Cancer and Leukaemia Group B. However, Japanese investigators reported a 23% response rate for the closely related irinotecan in patients with advanced breast cancer (Taguchi *et al*, 1994). The irinotecan schedules (based on weekly dosing) compared with the topotecan schedules (based on consecutive 5-day dosing) may, in part, explain the differences in overall response rates. By modifying the topotecan schedule to days 1 and 8, we more closely approximate the irinotecan administration schedule in our current study. The concordance between irinotecan and topotecan observed in our parallel analyses suggest that future *ex vivo* analyses could compare topotecan with other topoisomerase I inhibitors (e.g. 9-aminocamptothecin or 9-nitrocamptothecin).

Several topotecan combinations demonstrated synergy in NSCLC specimens, including topotecan/mitomycin-C (38%) and topotecan/cisplatin (31%). Both combinations revealed favourable *Z*-scores. Preliminary results for topotecan/cisplatin in NSCLC patients have provided overall response rates ranging from 14 to 31% (Raymond *et al*, 1997; Wiesenfeld *et al*, 1997). The high incidence of haematologic and nonhaematologic toxicity limited the use of this combination. Alternate dosing schedules for topotecan/cisplatin, or the substitution of carboplatin for cisplatin, might prove more tolerable. A phase II study of topotecan/carboplatin in NSCLC patients provided a clinical benefit in 49% of patients and a 1-year survival rate of 32% (Pujol *et al*, 2001). No episodes of nephrotoxicity, ototoxicity, or neurotoxicity were reported. Although synergy was demonstrated in <20% of specimens in NSCLC for topotecan/vinorelbine, the *Z*-scores for this combination, and those for topotecan/gemcitabine, fell within the sensitive range. Vinorelbine provides single-agent activity with overall response rates ranging from 12 to 20% in NSCLC (Crawford *et al*, 1996; The Elderly Lung Cancer Vinorelbine Italian Study Group, 1999). The *ex vivo* results revealing activity for topotecan/vinorelbine in NSCLC are consistent with the report of Stupp *et al* (2001), which provided a response rate of 42% in NSCLC patients treated with this combination

*Ex vivo* activity profiles also provide the opportunity to develop novel triplet regimens. The favourable activity observed for the doublets of topotecan plus cisplatin and topotecan plus gemcitabine paralleled our *ex vivo* results for the three-drug combination in NSCLC specimens (unpublished observations). Guarino *et al* (2002) reported a 38% objective response rate and 1-year survival of 33% in patients with NSCLC who received this weekly regimen. Toxicity was extremely mild with only one of 30 patients experiencing a grade IV adverse event (leukopenia). A second phase II trial is under final development to further evaluate this triplet in NSCLC (phase II study of hycamtin, cisplatinum, and gemcitabine in advanced NSCLC).

Several other observations were of interest. Despite the low single-agent activity of topotecan in prostate cancer specimens, the favourable degrees of synergy and activity for topotecan/cisplatin in this disease could provide insights for future investigation. On the contrary, the high degree of synergy for topotecan/mitomycin-C in colon cancer specimens when combined with the resistant *Z*-score for this combination in colon specimens argues against clinical evaluation. Although single-agent topotecan is widely used for the treatment of relapsed ovarian cancer, combinations identified in this study may offer additional insights. The high degree of synergy that we reported previously for topotecan/gemcitabine in ovarian cancer specimens (Su *et al*, 2001) is supported by the results of a phase II study, in which seven of 11 (64%) patients with relapsed ovarian cancer achieved objective responses with this combination (Sehouli *et al*, 2001). Topotecan combinations might also be investigated in diseases that are historically resistant to chemotherapy. Fiorica *et al* (2002) reported a 28% (nine of 32) overall response rate for topotecan/cisplatin in patients with recurrent cervical cancer.

In summary, topotecan reveals activity in human NSCLC and in breast, prostate, and colon cancer primary cultures. Previously, unrecognised synergy between topotecan and other agents have allowed the development of novel phase II clinical trials in NSCLC and breast cancers. Trials are also in development to evaluate topotecan in cervical, colorectal, prostate, and haematologic malignancies.
